# RNA-Seq Transcriptome Analysis of Rice Primary Roots Reveals the Role of Flavonoids in Regulating the Rice Primary Root Growth

**DOI:** 10.3390/genes10030213

**Published:** 2019-03-13

**Authors:** Yu Xu, Junjie Zou, Hongyan Zheng, Miaoyun Xu, Xuefeng Zong, Lei Wang

**Affiliations:** 1 College of Agronomy and Biotechnology, Southwest University, Chongqing 400716, China; xy13101391509@163.com; 2 Biotechnology Research Institute, Chinese Academy of Agricultural Sciences, Beijing 100081, China; zhenghongyan@caas.cn (H.Z.); xumiaoyun@caas.cn (M.X.)

**Keywords:** Quercetin, root growth, rice, RNA-seq, ROS, auxin

## Abstract

Flavonoids play important roles in root development and in its tropic responses, whereas the flavonoids-mediated changes of the global transcription levels during root growth remain unclear. Here, the global transcription changes in quercetin-treated rice primary roots were analyzed. Quercetin treatment significantly induced the inhibition of root growth and the reduction of H_2_O_2_ and O_2_^−^ levels. In addition, the RNA-seq analysis revealed that there are 1243 differentially expressed genes (DEGs) identified in quercetin-treated roots, including 1032 up-regulated and 211 down-regulated genes. A gene ontology (GO) enrichment analysis showed that the enriched GO terms are mainly associated with the cell wall organization, response to oxidative stress, and response to hormone stimulus. The Kyoto Encyclopedia of Genes and Genomes (KEGG) enrichment pathway analysis showed that the enriched DEGs are involved in phenylpropanoid biosynthesis, glutathione metabolism, and plant hormone signal transduction. Moreover, the quercetin treatment led to an increase of the antioxidant enzyme activities of catalase (CAT), peroxidase (POD), and superoxide dismutase (SOD) in rice roots. Also, the quercetin treatment altered the *DR5*:*GUS* expression pattern in the root tips. All of these data indicated that the flavonoids-mediated transcription changes of genes are related to the genes involved in cell wall remodeling, redox homeostasis, and auxin signaling, leading to a reduced cell division in the meristem zone and cell elongation in the elongation zone of roots.

## 1. Introduction

Flavonoids are a large group of secondary metabolites and are widely distributed throughout plants. The flavonoids are synthesized through the phenylpropanoid pathway, and can be classified into six major subgroups, including chalcones, flavonols, flavones, flavandiols, anthocyanines, and proanthocyanins, or condensed tannins [1,2]. In Arabidopsis, the flavonoid biosynthesis is directly regulated by several key enzymes, and is finely controlled by multiple transcription factors [3,4]. Flavonoids are considered as effective compounds involved in protecting plants against UV-B radiation [5], and as antioxidants to reduce the reactive oxygen species (ROS) levels in plants [6]. Additionally, flavonoids also function as important regulators in different biological processes, including defense against pathogen infection, nodulation, pollen fertility, and stomatal movement [2,7,8]. The abundance of flavonoids can be rapidly and profoundly regulated in response to environmental stimulus, growth, and developmental signals. 

Roots are important plant organs, anchoring the plants in the soil, providing the plants with nutrients and water, serving as energy storage organs, and also sensing and responding to a changed environment [9,10]. Flavonoids have been reported to play important roles in regulating root growth and development. In Arabidopsis roots, major flavonoids, including naringenin chalcone, kaempferol, and quercetin, display tissue-specific patterns [11,12]. Arabidopsis seedlings treated with naringenin lead to a reduction in root growth and gravitropism [12]. The addition of flavonoids results in reduced root growth and root numbers during the in vitro root formation in *Medicago truncatular* [13] mutations in the genes involved in the flavonoid biosynthetic pathway lead to an alteration in flavonoid accumulation patterns, and subsequently affect multiple aspects of plant architecture, including root development [12,14]. Arabidopsis *transparent testa4* (*tt4*) plants with a mutation in the gene encoding the first enzyme chalcone synthase (CHS) in flavonoid biosynthesis have no flavonoid accumulation, leading to increased secondary root development [15]. The *tt7* plants with a mutation in the flavonoid 3′-hydroxylase gene (*F3*′*H*) accumulate excess kaempferol, and the *tt3* plants with a lesion in the gene encoding dihydroflavonol reductase (DFR) accumulate excess kaempferol and quercetin [12]. The *tt3* and *tt7* mutants have disturbed root growth and root gravitropic responses [16]. Sweet potato transgenic plants’ heterologously expressed maize *Lc* gene has an increased anthocyanin pigment accumulation, which leads to a reduced size of the early storage roots [17]. In tomatoes, the *anthocyanin reduced (are)* plants with a mutation in the gene encoding flavonoid 3-hydroxylase (F3H) accumulate higher concentration of naringenin, displaying a reduced initiation of lateral roots and an increased number of root hairs, whereas the *anthocyanin without (aw)* plants with a defect in the gene encoding DFR contain more flavonols and no anthocyanins, displaying increased lateral root information. These results indicate the positive roles of flavonols in the formation of lateral roots, and their negative roles in primary root hairs [14]. 

Studies on the mechanisms of flavonoids in root development revealed that flavonoids mediate root development through regulating auxin transport and accumulation in the root tips [15,18,19,20]. Flavonoids can direct PIN-FORMED (PIN)-mediated polar auxin fluxes during root gravitropic responses [21]. In tomatoes, the *are* plants with a reduced flavonol accumulation exhibit an enhanced root hair formation through regulating the ROS levels, and the *aw* plants with elevated flavonols display an increased lateral root formation through modulation of the auxin transport [14]. Flavonoids also mediate the root growth direction through integrating hormonal and reactive oxygen species pathways in response to light [22]. UV-B radiation-induced root bending occurred through a flavonoid-mediated auxin pathway [23]. So, flavonoids may affect root development and root tropic responses through the regulation of auxin signaling and ROS levels.

In addition to affecting the auxin transport, auxin accumulation, and ROS levels, flavonoids may also function in regulating gene expression levels during root development. In Arabidopsis, the tissue-specific *PIN* expression levels are altered in the roots of the *tt3* and *tt4* mutants [18]. Flavonoid mutants with altered ROS scavenging abilities show altered gravitropic rates and 2-oxindole-3-acetic acid (oxIAA) accumulation, and oxIAA is inactive and does not induce the expression of the auxin-responsive reporter *DR5* [24]. In Arabidopsis seedlings, both quercetin and kaempferol have nuclear localization [12], indicating that the flavonoids may act as transcriptional regulators in the control of the transcription of the genes required for growth and development [25,26]. In sorghum roots, exogenous flavonoids inhibit primary root growth and increase the development of lateral roots through changing the expression patterns of specific genes *SHORT-ROOT* and *HD-ZIP III*, which are associated with root tissue differentiation [27]. Flavonoids have the potential to directly affect signaling and gene transcription through interactions with cytoplasmic or nuclear proteins, and they are capable of acting as transcriptional regulators in the control of the transcription of the genes required for growth and development [6,25,28]. However, whether flavonoids-mediated transcription changes at a whole genome level during root development remains unclear. 

Here, we conducted a RNA-seq of rice roots treated with quercetin so as to analyze the changes of the gene expression patterns involved in the flavonoid-mediated root development. The gene ontology (GO) enrichment analysis showed that the enriched DEGs are associated with cell wall organization or biogenesis, response to oxidative stress, and response to hormone stimulus under biological process GO terms. The Kyoto Encyclopedia of Genes and Genomes (KEGG) enrichment pathway analysis revealed that the enriched DEGs are mainly involved in phenylpropanoid biosynthesis, glutathione metabolism, and plant hormone signal transduction. Our results reported the differential gene expression patterns in the roots after treatment with quercetin, providing new insights into understanding flavonoids-mediated root development at a transcriptome level, mainly through controlling the cell wall dynamics, redox state, and plant hormone response.

## 2. Materials and Methods

### 2.1. Plant Materials and Growth Condition

The rice seeds used in this study were of a Nipponbare background (*Oryza sativa* L. *ssp. Japonica*). The rice seeds were surface sterilized with 70% ethanol for 2 min, washed with sterilized water, and then incubated in 2.5% NaClO for 30 min. After being washed with sterilized water three times, the seeds were then incubated in petri dishes in the dark for three days at 28 °C. The seedlings (the average primary root length was about 1.5 to 2 cm) were transferred and then incubated in the hydroponic culture with different concentrations of quercetin (0, 20, 50, and 100 μM). The seedlings were kept in a growth chamber with a 16-h light/8-h dark photoperiod at 25 °C. The culture solution was changed with a fresh solution every day. 

### 2.2. Measurement of Roots

To analyze the root meristem and elongation zone, the roots were incubated in a basic solution (7% NaOH in 60% ethanol) overnight at 37 °C. Then, the roots were placed in a mounting solution (50% glycerol in 10% ethanol), and photographed with an Invitrogen EVOS XL microscope. The meristem size and cell numbers, and the cell length in the elongation zone were measured using ImageJ software (http://imagej.nih.gov/ij/). 

### 2.3. Histochemical Staining for ROS Detection of H_2_O_2_ and O_2_^−^

For the 3,3-diaminobenzidine (DAB) staining to detect H_2_O_2_, the roots were incubated in a DAB staining buffer (0.6 mg/mL DAB in 0.1 M phosphate buffer, pH 7.0) for 3 h at room temperature. For the nitro blue tetrazolium (NBT) staining to detect O_2_^−^, the roots were incubated in an NBT staining buffer (0.1 mg/mL NBT in 0.1 M phosphate buffer, pH 7.0) for 3 h at room temperature. Both the DAB staining buffer and NBT staining buffer were freshly prepared. The samples stained with DAB or NBT were then washed twice with a 0.1 M phosphate buffer, stored in 70% ethanol, and photographed using a stereoscope (M165 FC, Leica). The intensity of the staining signals was measured using ImageJ software (http://imagej.nih.gov/ij/).

### 2.4. RNA Sequencing (RNA-seq) and Data Analysis

After treatment with 0 or 50 μM quercetin for two days, about 50 roots of each sample were excised and frozen immediately in liquid nitrogen. Then, the samples were sent to Biomarker Biotechnology Corporation (Beijing, China) for RNA-seq library preparation. Three biological replicates were used for RNA-seq experiments. A total amount of 1 μg RNA per sample was used as the input material for the RNA sample preparations. Sequencing libraries were generated using a NEBNext Ultra^TM^ RNA Library Prep Kit for Illumina (NEB, USA), following manufacturer’s recommendations. The library quality was assessed on the Agilent Bioanalyzer 2100 system. The clustering of the index-coded samples was performed on a cBot Cluster Generation System using a TruSeq PE Cluster Kit v4-cBot-HS (Illumia), according to the manufacturer’s instructions. After cluster generation, the library preparations were sequenced on an Illumina platform, and paired-end reads were generated. The raw data were submitted to CNGB (China National Genebank) Nucleotide Sequence Archive (CNSA) (http://db.cngb.org/cnsa/), and the accession code was CNP0000347.

A differential expression analysis was performed using DESeq R package (1.10.1). The resulting *p*-values were adjusted using the Benjamini and Hochberg’s approach for controlling the false discovery rate (FDR). The genes with a significant threshold FDR value of <0.01, and an absolute log_2_ fold change of >1 were assigned as being differentially expressed [29].

For the GO enrichment analysis, a Singular Enrichment Analysis (SEA) tool was performed in AgriGO v2.0, with default parameters set and a threshold FDR adjusted *p*-value of <0.05. Rice MSU 7.0 (TIGR) was used as the background or reference. [30]. The statistical enrichment of the DEGs in the KEGG pathway was conducted using KOBAS software [31]. The MapMan tool (http://MapMan.gabipd.org) was used to visualize the involvement of the DEGs in the pathways [32].

### 2.5. RNA Extraction and Real-Time Quantitative PCR (qRT-PCR)

After treatment with 0, 50, or 100 μM of quercetin, more than 50 roots of each sample were excised and collected for RNA extraction. The total RNA was extracted using the TRNzol reagent (DP405-02, Tiangen, China). After removing the DNA with DNase (Promega, M6101), the RNA was reversed transcribed using a GoScript^TM^ Reverse Transcription System (Promega, M6101A5001). For the quantitative real-time PCR (qRT-PCR), a 2 × SYBR^®^ Green Master Mix (Takara, Japan) was added to the reaction mix and run on an Applied Biosystems 7500 Real Time PCR System (Applied Biosystems, Foster City, CA, USA). For each gene, the measurements were performed in four replicates, and the average cycle thresholds (Ct) were used to determine the fold-change. Rice ACTIN1 (LOC_Os03g50885) was used as the reference control. The results were calculated using the 2^−∆∆Ct^ method [33]. All of the primers used for the qRT-PCR are listed in Table S1.

### 2.6. Assays of Superoxide dismutase (SOD), Peroxidase (POD), and Catalase (CAT)Activities

After treatment with 0, 50, or 100 μM of quercetin for two days, the roots excised from the seedlings were immediately frozen in liquid nitrogen and were finely ground into powder with a pestle. Then, 100 mM phosphate buffer (pH 7.0) was added, and the final concentration of the samples was 10% (*w*/*v*). The samples were then vortexed and centrifuged at 3500 × *g* rpm for 10 min at 4 °C. The supernatants were collected and the protein content was analyzed with bicinchoninic acid (BCA) kit (Kangwei, Beijing, China, CW0014). The activities of CAT, SOD, and POD were determined according to the protocols of the catalase assay kit (A007-1-1), total superoxide dismutase (T-SOD) assay kit (A001-1-1), and plant peroxidase assay kit (A084-3), respectively, obtained from Nanjing Jiancheng Bioengineering Institute (Nanjing, China). 

### 2.7. DR5-GUS Staining

After growth for three days, the seedlings were then treated with a different concentration of quercetin for another two days. The seedlings were stained with a X-Gluc solution for 12 h. Then, the roots were mounted and photographed using a stereoscope (M165 FC, Leica).

## 3. Results

### 3.1. Treatment with Quercetin Inhibited Rice Root Growth

**Figure 1:**
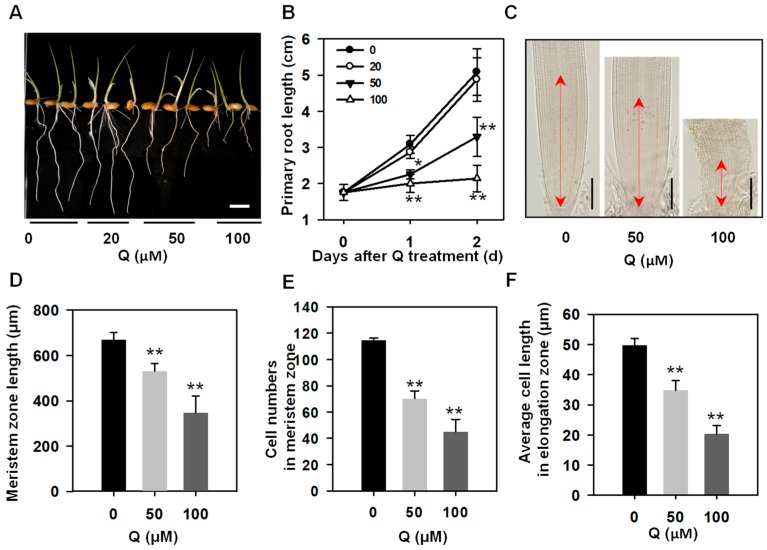
The effect of quercetin on rice root growth. (**A**) The rice root growth phenotype with 0, 20, 50, or 100 μM of quercetin (Q) treatment. Photos were taken after treatment with different concentration of quercetin for two days. Bar = 1 cm. (**B**) Time course of the primary root length of the rice seedlings treated with quercetin. Values are means ± standard deviation (SD; *n* = 60). The asterisks indicate a significant difference between the control and quercetin-treated plants at an indicated time (Student’s *t* test, * *p* < 0.05, ** *p* < 0.01). (**C**) Size of the root meristem with 0, 50, or 100 μM of quercetin treatment for two days. Double-headed arrow indicates the meristem zone. Bars = 200 μM. (**D**) The meristem zone length with 0, 50, or 100 μM quercetin treatment for two days. Values are means ± SD (*n* = 10). (**E**) The cell numbers in the meristem zone with 0, 50, or 100 μM quercetin treatment for two days. Values are means ± SD (*n* = 10). (**F**) Average cell length in the elongation zone with 0, 50, or 100 μM quercetin treatment for two days. Values are means ± SD (*n* = 10). The asterisks in (**D**–**F**) indicate a significant difference between the control and quercetin-treated plants (Student’s *t* test, ** *p* < 0.01).

To analyze the effects of the flavonoids on the rice root growth at transcriptional levels, quercetin, a major kind of flavonol, was used in this study. The rice seedlings were treated with a different concentration of quercetin, and the primary root phenotypes were analyzed firstly. After germination for two days, the rice seedlings were treated with 20, 50, or 100 μM quercetin for another two days. As shown in Figure 1A, the lengths of the rice primary roots treated with 20 μM of quercetin were similar to those of the untreated plants. However, the lengths of the rice primary roots treated with 50 or 100 μM quercetin were significantly shorter than those of the untreated plants, displaying a dose-dependent manner (Figure 1A). The analysis of the root growth rates showed that the rice primary roots treated with 50 or 100 μM of quercetin displayed a reduced root growth rate compared with the untreated plants, and the 20 μM quercetin-treated rice primary roots showed a similar root growth rate as the untreated plants (Figure 1B). To determine whether the root growth defects arose from a defective cell proliferation or cell elongation, we compared the root meristem zone and elongation zone between the untreated plants and quercetin-treated plants. The measurement of the root meristem length showed that the meristem size in the 50 or 100 μM quercetin-treated root tips was significantly shorter than that in the untreated root tips (Figure 1C,D). Also, the 50 or 100 μM quercetin-treated plants had less cell numbers in the root meristem zone than the untreated plants (Figure 1E). The average cell length in the elongation zone of the roots of the 50 or 100 μM quercetin-treated seedlings was shorter than that in the untreated plants (Figure 1F). These results indicated that quercetin inhibits rice root growth through reducing the root meristem zone size and cell elongation in the elongation zone.

### 3.2. Treatment with Quercetin Reduced ROS Levels in the Root Tips

**Figure 2:**
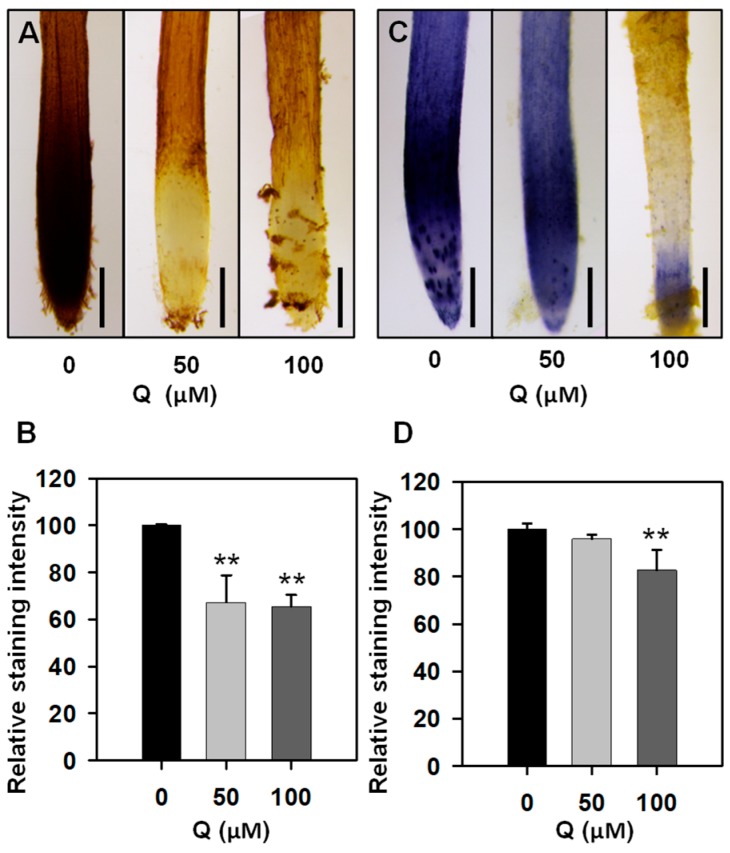
Reactive oxygen species (ROS) accumulation in the primary root tips with or without quercetin treatment. (**A**) The 3,3-diaminobenzidine (DAB) staining for H_2_O_2_ in the rice primary root tips with or without quercetin treatment. Experiments were repeated three times with similar results. Three to six roots were used for each experiment. Bars = 200 μm. (**B**) The DAB staining intensity in the rice primary root tips was determined with ImageJ. (**C**) NBT staining for O_2_^−^ in rice primary root tips with or without quercetin treatment. Experiments were repeated three times with similar results. Three to six roots were used for each experiment. Bars = 200 μm. (**D**) The nitro blue tetrazolium (NBT) staining intensity in the rice primary root tips was determined with ImageJ. The asterisks in (**B**,**D**) indicate the differences between the untreated and quercetin-treated rice root tips (Student *t* test, ** *p* < 0.01).

Previous reports revealed that flavonoids act as antioxidants to scavenge ROS [6,34,35]. To test whether the ROS levels can be affected in the quercetin-treated plants, we then used DAB and NBT staining methods to detect the H_2_O_2_ and O_2_^−^ levels in the primary root tips, respectively. After staining with a DAB solution, the staining intensity in the 50 or 100 μM quercetin-treated root tips was significantly lower compared with the untreated rice roots (Figure 2A,B). The NBT staining results showed that the quercetin treatment could significantly reduce the O_2_^−^ production, and the NBT staining intensity in 100 μM quercetin-treated root tips was much lower than that in the 50 μM quercetin-treated root tips (Figure 2C,D). These results indicated that the quercetin treatment could reduce both the H_2_O_2_ and O_2_^−^ accumulations, affecting the root meristem activity and cell elongation.

### 3.3. The Enriched GO Terms were Mainly Associated with Cell Wall Organization, Oxidative Stress, and Plant Hormone Signal Transduction

To get global insights into the DEGs related to the flavonoids inhibition of root growth, rice primary roots treated with or without 50 μM of quercetin were collected for RNA extraction and sequencing. Three biological replicates were conducted and their repeatability was assayed by calculating the Pearson correlation coefficient. The results were consistent and repeatable. The DEGs were identified between the control and quercetin treatment samples using DESeq. After treatment with 50 μM quercetin for two days, 1243 DEGs were identified, including 1032 up-regulated and 211 down-regulated genes (Table S2). 

**Figure 3:**
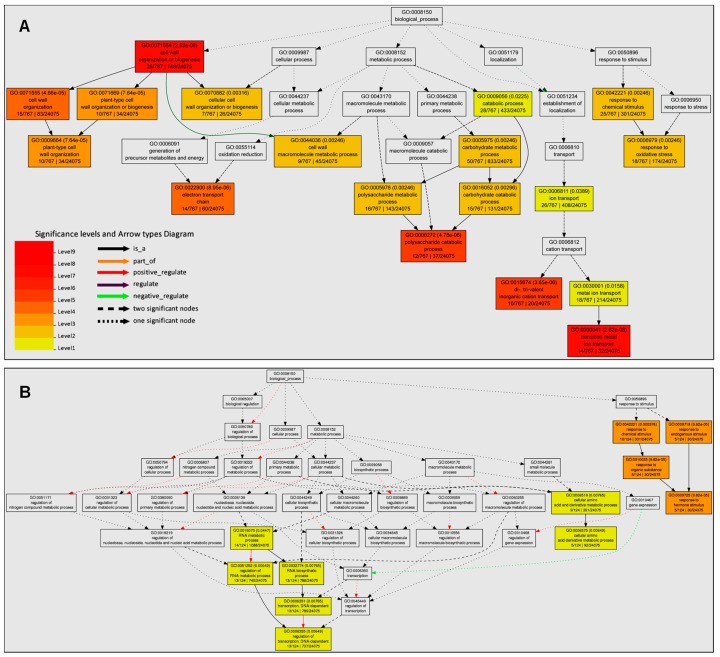
Gene ontology (GO) enrichment analysis of DEGs in the biological process GO term using AgriGO v2. (**A**) GO enrichment analysis of up-regulated DEGs. (**B**) GO enrichment analysis of down-regulated DEGs. Singular enrichment analysis (SEA) was performed in AgriGO v2 in order to identify the enriched GO terms for up-regulated or down-regulated genes. Each box shows the GO term number, the *p*-value, and GO term. The box color indicates the level of statistical significance.

To classify the biological function of the DEGs, a GO enrichment analysis was carried out using the Singular Enrichment Analysis (SEA) tool offered by AgriGO V2 [30]. The background query list contained 24,075 annotated genes from MSU 7.0 version (http://rice.plantbiology.msu.edu/). The GO enrichment analysis of the 767 annotated up-regulated genes identified 60 significantly (FDR <0.05) enriched GO terms for the biological process, cellular component, and molecular function categories (Table S3). Within the biological process category, the enriched DEGs were mainly associated with the plant-type cell wall organization (GO:0009664), transition metal ion transport (GO:0000041), polysaccharide metabolic process (GO:0005976), and the response to oxidative stress (GO:0006979) (Figure 3A, Table S3). Within the cellular component category, the enriched DEGs were mainly associated with the extracellular region part (GO:0044421), membrane (GO:0016020), membrane-bounded organelle (GO:00432227), and mitochondrion (GO:0005739) (Figure S1A, Table S3). Within the molecular function category, the DEGs were associated with the transferase activity (GO:0016765), electron carrier activity (GO:0009055), chitinase activity (GO:0004553), and peroxidase activity (GO:0004601) (Figure S1B, Table S3). For the 124 annotated down-regulated genes, there were 24 significantly enriched GO terms for the biological process and cellular component categories, and no down-regulated DEGs were enriched within the molecular function category (Table S4). Within the biological process category, the enriched DEGs were mainly associated with the response to the hormone stimulus (GO:0009725) (Figure 3B). Within the cellular component category, the enriched DEGs were associated with the plastid (GO:0009536) and mitochondrion (GO:0005739) (Figure S2). 

We then further analyzed the DEGs involved in the enriched biological process GO terms for up-regulated and down-regulated genes. For “plant-type cell wall organization (GO:0009664)”, all of the genes encode expansins (Table S5). For “polysaccharide catabolic process (GO:0000272)”, most of the genes participate in regulating the chitin activity or cellulose activity (Table S5). The plant cell wall as an extracellular matrix or region is a dynamic structure that plays numerous roles in the physical control of growth, the establishment of the cell shape, and the maintenance of the structural integrity of the plant body in response to environmental cues [36]. The enriched GO terms involved in the cell wall organization indicated that quercetin functions in regulating the cell wall remodeling and the subsequent cell elongation. For “response to oxidative stress (GO:0006979)”, 16 of the 18 genes encode peroxidases, indicating that quercetin participates in regulating the expression pattern of antioxidant genes, especially the peroxidase genes (Table S5). The genes for “response to hormone stimulus (GO:0009725)” contain Os11g0137000 (*OsPIN1B*), Os06g0166500 (*OsIAA20*), and Os05g0500900 (*OsGH3-4*), which are involved in the auxin transport and signaling (Table S5). These results indicate that quercetin functions in the root growth, mainly through regulating the transcription levels of the genes involved in cell wall remodeling, redox homeostasis, and auxin signaling.

### 3.4. KEGG Pathway Enrichment Analysis Revealed That the Enriched DEGs Are Involved in Phenylpropanoid Biosynthesis, Glutathione Metabolism, and Plant Hormone Signal Transduction

**Figure 4:**
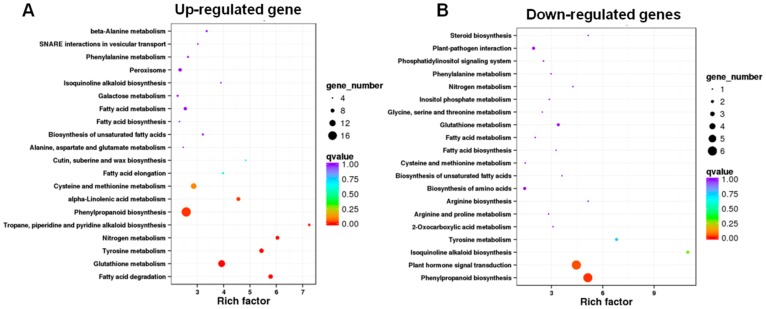
The Kyoto Encyclopedia of Genes and Genomes (KEGG) pathway enrichment scatter plot of DEGs. (**A**) KEGG pathway enrichment analysis for up-regulated genes. (**B**) KEGG pathway enrichment analysis for down-regulated genes. The *x*-axis indicates the rich factor, and the degree of KEGG pathway enrichment. The *y*-axis indicates the name of the KEGG pathway. The dot size means the gene number. The dot color indicates the *q*-value. The top 20 KEGG pathways are shown.

To further characterize the function of the DEGs in quercetin-mediated root growth, a pathway-based analysis was performed using the KEGG pathway database (https://www.genome.jp/kegg/). The KEGG enrichment analysis showed that the phenylpropanoid biosynthesis (23 genes, 17 up-regulated, and 6 down-regulated), glutathione metabolism (15 genes, 13 up-regulated, and 2 down-regulated), and plant hormone signal transduction (6 down-regulated) are enriched, indicating that quercetin functions in regulating root growth through regulating these genes’ expression patterns (Figure 4, Tables S6 and S7). In the “phenylpropanoid biosynthesis” pathway, most of the genes are peroxidase and glycosyl hydrolase family members (Tables S6 and S7), which participate in regulating the ROS levels and cell wall organization, leading to cell wall remodeling [37]. In the “glutathione metabolism” pathway, 12 of the total 15 genes encode glutathione S-transferases (GSTs) (Tables S6 and S7). GSTs in plants can protect tissues against oxidative damage because of their role in the enzymatic detoxification of endo- and xeno-biotics, controlling redox homeostasis [38]. In the “plant hormone signal transduction” pathway for down-regulated genes (Table S7), two genes, Os05g0500900 (*OsGH3-4*) and Os06g0166500 (*OsIAA20*), are involved in auxin signaling transduction. These findings indicate that quercetin regulates root growth mainly through regulating the expression levels of the genes involved in the phenylpropanoid biosynthesis, glutathione metabolism, and plant hormone signal transduction metabolic pathways. 

### 3.5. Metabolism Pathway Analysis of DEGs Using MapMan

**Figure 5:**
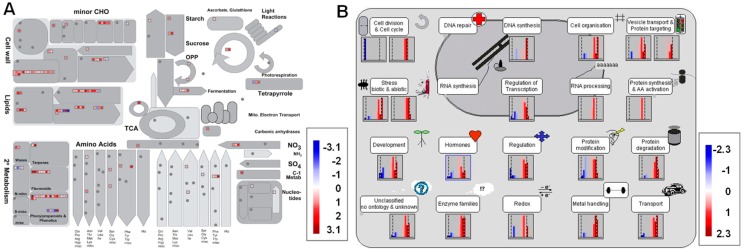
MapMan overview of the DEGs in the quercetin-treated rice roots. (**A**) The metabolism overview in MapMan. (**B**) The cell functions overview in MapMan. The DEGs were binned to the MapMan functional categories. The values are the log_2_ fold changes. The up-regulated and down-regulated genes are represented with red and blue squares, respectively. Details about the genes are shown in Table S8.

To further investigate the potential functions of the DEGs, we analyzed the metabolic processes and cell functions using the MapMan tool [32]. The DEGs in the cell walls, lipids, and secondary metabolisms were enriched in the metabolism pathway (Figure 5A, Table S8). Most of the DEGs in the cell wall metabolism pathway encode expansin, consistent with the GO enrichment analysis. For the second metabolism, the enriched DEGs were correlated to the flavonoids, phenylpropanoids and phenols, and lignin biosynthesis metabolism pathways (Figure 5A, Table S8). When analyzing the cell function overview in MapMan, the enriched DEGs were associated with biotic or abiotic stress, cell organization, vesicle transport and protein targeting, regulation of transport, redox, hormones, and so on (Figure 5B, Table S8). The MapMan analysis further validated the GO and KEGG enrichment analyses (Figure 3 and Figure 4).

### 3.6. Validation of RNA-Seq Data by qRT-PCR

**Figure 6:**
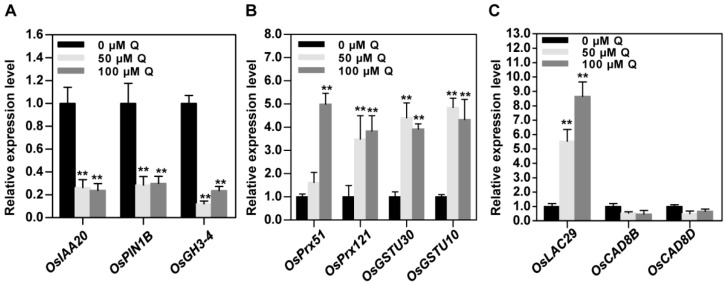
qRT-PCR-based identification of auxin, antioxidant, and cell wall-related DEGs from RNA-seq. (**A**) Validation of several genes involved in the auxin signaling with 0, 50, or 100 μM of quercetin (Q). (**B**) Validation of several genes involved in the antioxidant activity with 0, 50, or 100 μM of quercetin (Q). (**C**) Validation of several genes involved in cell wall dynamics with 0, 50, or 100 μM of of quercetin (Q). Values are means ± SD (*n* = 4). The asterisks indicate a significant difference relative to the untreated plants (Student’s *t* test, ** *p* < 0.01).

To confirm the results from the RNA-seq analysis, some DEGs in the enriched GO terms and KEGG pathways were selected, and their expression levels were confirmed by qRT-PCR analysis. Auxin regulates plant growth and development by altering the expression of diverse genes, including the auxin/indole-3-acetic acid (Aux/IAA), small auxin up-regulated RNA (SAUR), and Gretchen Hagen 3 (GH3) family members [39]. In addition, flavonoids can negatively regulate auxin transport by affecting the expression and protein localization of the PIN auxin efflux facilitators [18]. The rice genome contains 12 members of the GH3 gene family, and the transcript abundance of nearly all of the *OsGH3* genes is enhanced on the auxin treatment, especially *OsGH3-1*, *-2,* and *-4* [39]. We then selected Os06g0166500 (*OsIAA20*), Os11g0137000 (*OsPIN1B*), and Os05g0500900 (*OsGH3-4*), and their expression levels were analyzed by qRT-PCR. The qRT-PCR results showed that the transcription levels of these genes were down-regulated by quercetin treatment, consistent with the RNA-seq results (Figure 6A). These results indicated that quercetin mediates the gene expression levels involved in regulating the auxin metabolism, auxin transport, and auxin signal transduction.

Another group of genes involved in the antioxidant systems include the peroxidase and glutathione S-transferase genes. The RNA-seq results show that most of these genes are up-regulated after quercetin treatment. Here, two peroxidase genes, *Os03g0762300* (*OsPrx51*) and *Os09g0323700* (*OsPrx121*), and two glutathione S-transferase genes, *Os10g0529500* (*OsGSTU30*) and *Os10g0531400* (*OsGSTU10*), were selected for the qRT-PCR analysis. Their transcription levels were induced after quercetin treatment, consistent with the RNA-seq results (Figure 6B). Peroxidase and glutathione S-transferase are important for controlling the ROS level and redox homeostasis. The quercetin treatment induced the increased expression levels of these antioxidant system-related genes, and then improved the enzyme activities so as to reduce the ROS levels in the plants.

In the extracellular matrix or region, the cell wall proteins are important for controlling cell growth, cell shape, and structural integrity [36]. Here, Os12g0258700 (*OsLAC29*), Os09g0400000 (*OsCAD8B*), and Os09g0400400 (*OsCAD8D*) were selected for the qRT-PCR analysis. *OsLAC29* encodes a laccase, which belongs to a member of the multicopper oxido-reductases. The *OsLAC29* expression level was increased after quercetin treatment, consistent with the RNA-seq results (Figure 6C). *OsCAD8B* and *OsCAD8D* encode cinnamyl alcohol dehydrogenases, which are involved in the lignin biosynthesis [17]. Their expression levels were not significantly altered after treatment with quercetin. 

### 3.7. Quercetin Treatment Enhanced the Activities of Antioxidant Enzymes 

**Figure 7:**
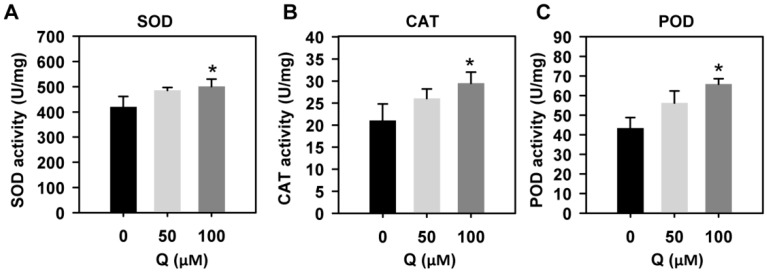
SOD, CAT, and POD activities in rice roots treated with or without quercetin. (**A**) SOD activities in rice roots treated with 0, 50, or 100 μM of quercetin. (**B**) CAT activities in rice roots treated with 0, 50, or 100 μM of quercetin. (**C**) POD activities in rice roots treated with 0, 50, or 100 μM of quercetin. Three-day-old rice seedlings were treated with 0, 50, or 100 μM of quercetin for another two days. The roots were excised from the seedlings and the total proteins were prepared for the SOD, CAT, and POD assays. The data are presented as means ± SD (*n* = 3). Asterisks indicate the significant difference relative to WT (Student’s *t*-test, * *p* < 0.05).

Enzymatic antioxidants, including CAT, SOD, and POD, are important ROS scavenging enzymes in reducing ROS levels. We then analyzed the antioxidant activities of SOD, POD, and CAT in the roots, with or without quercetin treatment. After treatment with quercetin, the activities of SOD, CAT, and POD in the roots were increased and were higher than those in the untreated roots (Figure 7). These results indicated that the flavonoids function in regulating the ROS scavenging enzyme activities. 

### 3.8. DR5:GUS Expression Pattern in the Roots Was Altered after Quercetin Treatment

**Figure 8:**
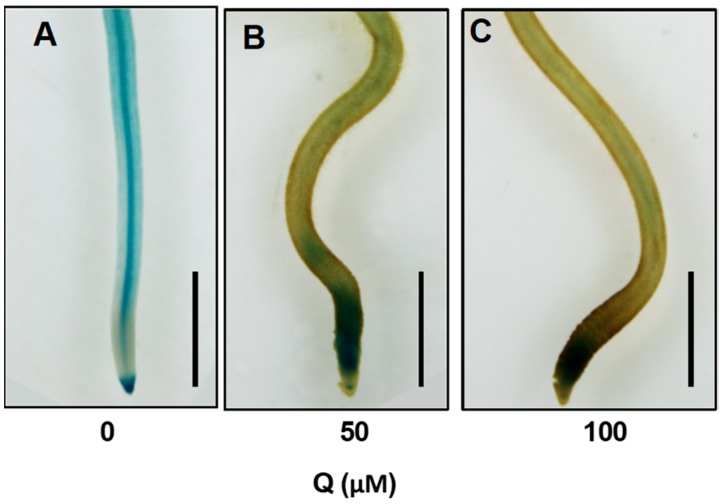
Quercetin treatment affects *DR5:GUS* expression patterns. Three-day-old *DR5:GUS* transgenic rice seedlings grown in a liquid medium were treated with quercetin for another two days. The roots were then stained in a X-gluc buffer for 12 h at 37 °C in the dark. The *DR5:GUS* expression patterns of the untreated (**A**), 50 μM of quercetin (**B**), and 100 μM of quercetin (**C**) plants are presented. Bars = 1 mm.

To further analyze the role of quercetin on plant hormone response, we used the *DR5:GUS* transgenic rice line to assay the effect of quercetin on the *DR5:GUS* expression pattern in the root tips. After growth for three days, the *DR5:GUS* plants were treated with 50 or 100 μM of quercetin for another two days. The seedlings were then used for the GUS staining assay. As shown in Figure 8, the *DR5:GUS* roots treated with quercetin had a different GUS staining pattern compared with the untreated rice roots. A higher GUS staining intensity was accumulated in the root tips, whereas there were weak GUS staining signals detected in the mature zone of the roots. These results indicate that the quercetin treatment may affect auxin transport and accumulation in the roots. 

## 4. Discussion

Flavonoids act as important regulators in primary root growth and development, as well as in root direction growth in response to light and gravitropic stimulation [14,15,17,19,21,22,23]. Flavonoids have the ability to modulate the signaling cascades that regulate cell growth and differentiation, whereas the functional significance has not yet been explored in detail [26]. Here, our results show that quercetin treatment significantly inhibits primary root growth and reduces ROS levels in rice roots (Figure 1 and Figure 2). Then, the RNA-seq of the rice primary roots with or without quercetin treatment was conducted in order to analyze the flavonoid-mediated transcriptome changes during root growth. The GO, KEGG, and MapMan analyses indicated that the enriched DEGs in the quercetin-treated roots are involved in cell wall remodeling, redox homeostasis, and plant hormone signal transduction (Figure 3, Figure 4 and Figure 5). Our results provided new insights into understanding the mechanism of the flavonoids in primary root growth at a global transcriptome level.

Flavonoids as antioxidants can regulate the ROS levels and modulate root growth and development [14]. H_2_O_2_ and O_2_^−^ have different distribution patterns in the root tips, and the balance of H_2_O_2_ and O_2_^−^ between the zones of cell proliferation and the zone of cell elongation controls the transition from proliferation to differentiation, regulating the root growth and differentiation [37,40]. Rice seedlings with reduced ROS levels in the root tips have a short root phenotype and reduced meristem activity [41]. Here, the exogenous application of quercetin significantly inhibited the primary root growth of rice, displaying a short root length, reduced cell number in the meristem zone, and cell length in the elongation zone (Figure 1), similar to a previous report about Arabidopsis plants with accumulated flavonoids [42]. Both the H_2_O_2_ and O_2_^−^ levels in the quercetin-treated roots were much lower than those in the untreated roots (Figure 2). The RNA-seq analysis showed that some enriched up-regulated DEGs are associated with the peroxidase activity GO term (Figure 3A, Table S3). The quercetin treatment also changed the expression of the redox status-related genes, including glutathione S-transferases (GSTs), and most of these genes were induced by quercetin treatment (Table S6). GSTs constitute a family of enzymes best known for their role in the enzymatic detoxification of endo- and xeno-biotics [38]. Also, higher activities of antioxidant enzymes (including CAT, SOD, and POD) were found in the quercetin-treated roots compared with the control (Figure 7). These results indicate that flavonoids and the increased expression levels of redox and ROS scavenging-related genes contribute to the reduction of ROS levels in roots. On the other hand, some transcription factors are both direct and indirect targets of redox-dependent activity modulation (Table S8) [43]. The redox-control of the transcription factors will regulate the transcription levels of the genes in rice development.

Flavonoids can act as direct or indirect inhibitors of auxin transporters [18,44], and function in altering the auxin movement and metabolism [24], modulating auxin transport, accumulation, and signaling. Flavonoid mutants with altered levels of quercetin and kaempferol have a different expression and protein localization of the PIN proteins [18]. Here, our results show that quercetin treatment inhibits the expression levels of the genes involved in auxin response (*OsIAA20* and *OsGH3-4*) and transport (*OsPIN1B*) (Figure 6A). So, quercetin treatment altered the auxin transport and accumulation, as well as the auxin responsive gene expression levels, leading to a change of auxin metabolism and signaling. The altered *DR5:GUS* expression pattern in the quercetin-treated roots also confirmed the role of quercetin in auxin signaling (Figure 8). 

Previous reports revealed that flavonoids regulate plant growth and development through the regulation of the auxin transport and cellular redox status [45]. Flavonoids can inhibit the auxin transport and reduce the ROS levels in the roots [22,23]. Auxin can induce ROS production, and flavonoids also influence auxin signaling by buffering the ROS formation and ROS-dependent auxin oxidation [24]. Auxin and ROS could also induce flavonoid production in the roots [22,46]. These findings reveal the complex regulatory mechanism of flavonoid-mediated root growth. The flavonol levels in the roots are therefore finely and tightly controlled for normal root growth, and in response to environmental stimulus.

The GO enrichment analysis showed that there are 10 enriched DEGs (encoding expansins) in the plant-type cell wall organization GO term, which take part in the cell wall dynamics (Figure 3A, Table S5). The cell expansion is tightly associated to the cell wall loosening and stiffening during plant growth. The KEGG metabolism pathway analysis shows that most of the enriched differentially expressed genes in the phenylpropanoid biosynthesis pathway encode the peroxidases and glycosyl hydrolases (Table S6). Glycosyl hydrolases have been implicated in diverse roles, including the lignification and cell wall remodeling [47]. The cell wall class III peroxidases are capable of generating ROS, such as OH• and HOO•, but can also regulate the level of H_2_O_2_. Therefore, these genes play a critical role in cellular growth, by controlling the subtle balance between cell wall loosening and de novo cell wall synthesis/cell wall strengthening [36]. All of these genes are important for cell wall remodeling [47,48]. So, quercetin participated in regulating the transcription levels of the genes involved in cell wall remodeling, contributing to cell elongation. 

Flavonoids distribute to different cellular compartments [6], and may regulate the gene expression levels through interactions with cytoplasmic or nuclear proteins [28]. Flavonoids are capable of acting as transcriptional regulators in the control of the transcription of the genes required for growth and development [6,25,28]. In mammals, flavonoids may play important roles as signaling molecules, through their ability to interact with a wide range of proteins, including mitogen activated protein kinases (MAPKs) [49]. In plants, flavonoids may also interact with cytoplasmic- and nuclear-distributed MAPKs, affecting MAPK activities and the subsequent MAPK signaling cascade [26]. 

In Arabidopsis, flavonoids display tissue-specific accumulation patterns [11], and flavonoid mutants in the flavonoid biosynthetic pathway accumulate different kinds of flavonoids that have different subcellular localizations [12]. Flavonoid biosynthetic pathway mutants have different root architecture phenotypes and gravity responses, indicating the complexity of different kinds of flavonoids in regulating root development [16]. The functional analysis of the genes involved in rice flavonoid biosynthetic pathways will reveal their specific roles in root development, and elucidate the individual flavonoid-mediating signaling pathway. 

## 5. Conclusions

Flavonoids are required for root development and for its tropic response. Flavonoids have served as important regulators during root development, whereas the flavonoid-mediated transcriptome changes are still unclear in the roots. An increased accumulation of flavonoids, such as quercetin, affects root growth. To gain global insights into the molecular mechanism of the flavonoids in root growth, an RNA-seq of quercetin-treated rice roots was performed. Currently, our RNA-seq analysis showed that there are 1243 differentially expressed genes in quercetin-treated rice primary roots. The GO, KEGG, and MapMan analyses that the enriched DEGs are mainly involved in cell wall remodeling, redox homeostasis, and plant hormone signaling (Figure S3). Our results revealed that flavonoids act not only as ROS scavengers, but also as important signaling molecules in order to regulate the gene expression patterns. These results may provide further insights into understanding the importance of flavonoids in regulating rice root growth and development. 

## Supplementary Materials

Supplementary materials are available online at https://www.mdpi.com/2073-4425/10/3/213/s1. Figure S1: GO term enrichment analysis of up-regulated DEGs in cellular component and molecular function GO term. Figure S2: GO term enrichment analysis of down-regulated DEGs in cellular component GO term. Figure S3: A mode of flavonoids-mediated global transcription changes during rice root growth. Table S1: Primers used for qRT-PCR analysis. Table S2: DEGs in quercetin-treated roots. Table S3: GO enrichment analysis of up-regulated genes. Table S4: GO enrichment analysis of down-regulated genes. Table S5: Detailed gene information for enriched plant-type cell wall organization, polysaccharide catabolic process, response to oxidative stress, and response to hormone stimulus GO terms. Table S6: KEGG enrichment analysis of up-regulated genes. Table S7: KEGG enrichment analysis of down-regulated genes. Table S8: Detailed information for MapMan terms.
