# Supplementary figures and images for: RNA-Seq Transcriptome Analysis of Rice Primary Roots Reveals the Role of Flavonoids in Regulating the Rice Primary Root Growth
Source: Genes (Basel). 2019 Mar 13;10(3):213. doi: 10.3390/genes10030213 (PMC6470995; doi:10.3390/genes10030213)

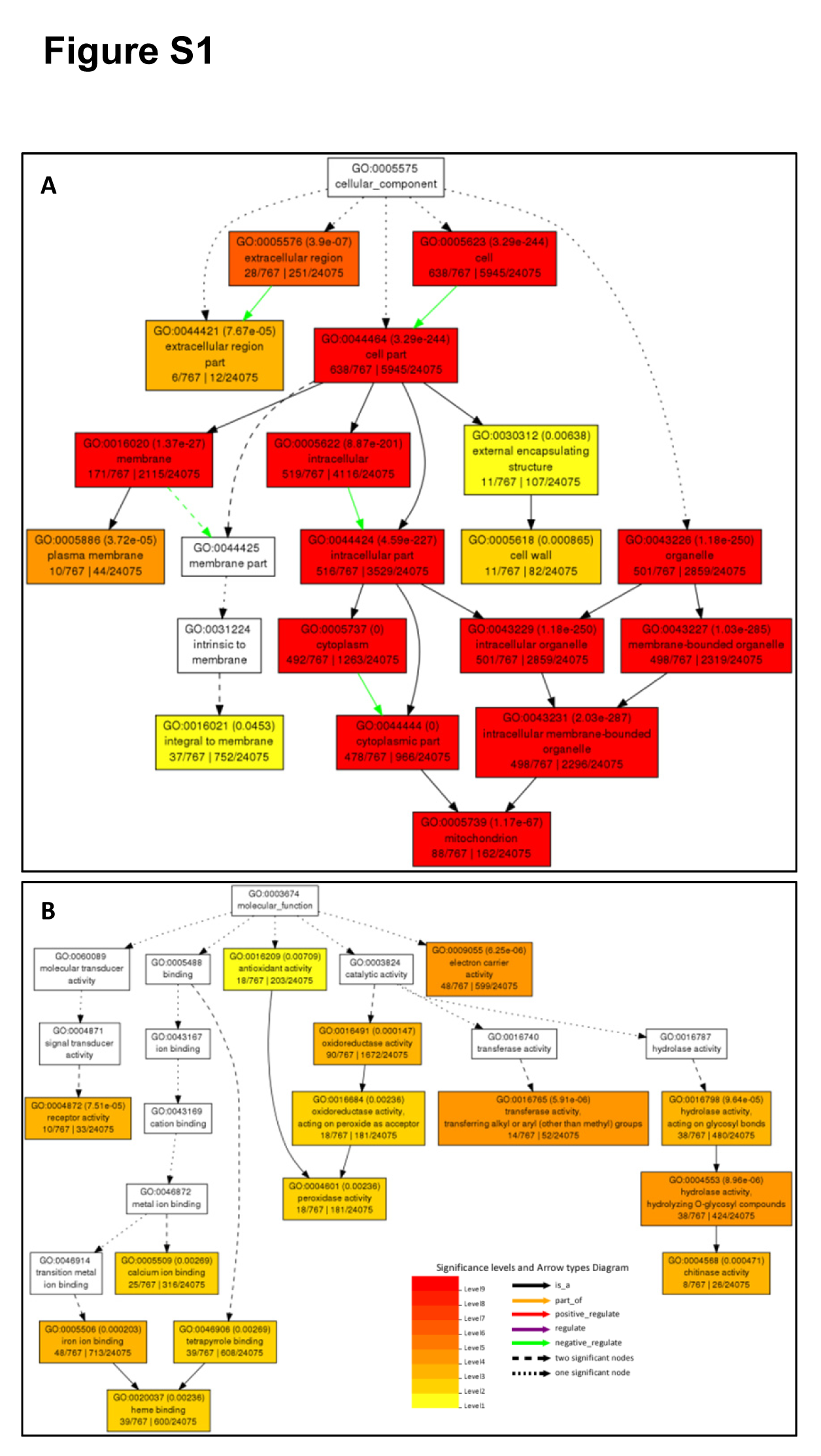

Supplement: Supplementary file 1 [file genes-10-00213-s001.zip › Xu et al-2019-Supplementary files v3/Figure S1.tif]

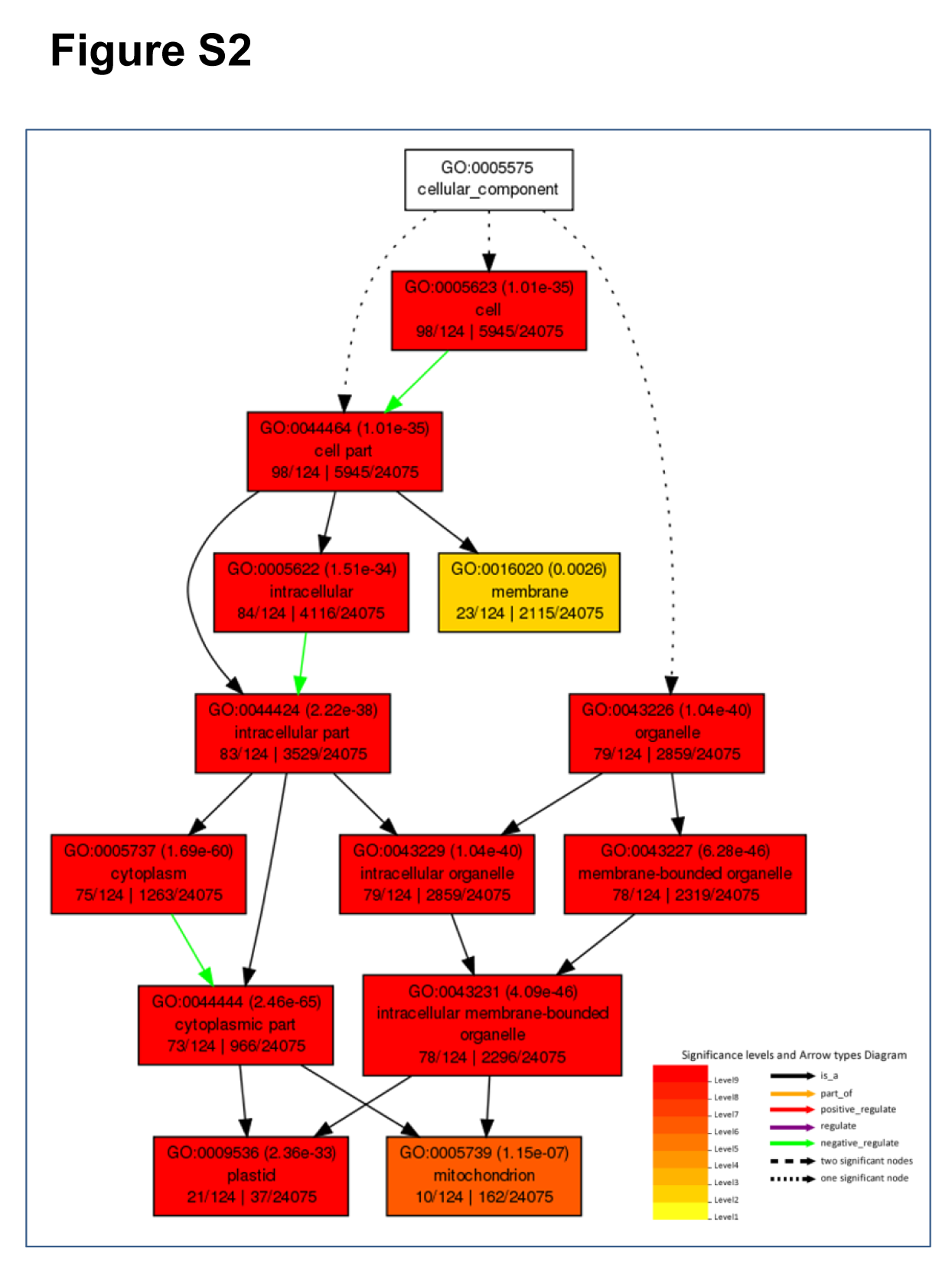

Supplement: Supplementary file 1 [file genes-10-00213-s001.zip › Xu et al-2019-Supplementary files v3/Figure S2.tif]

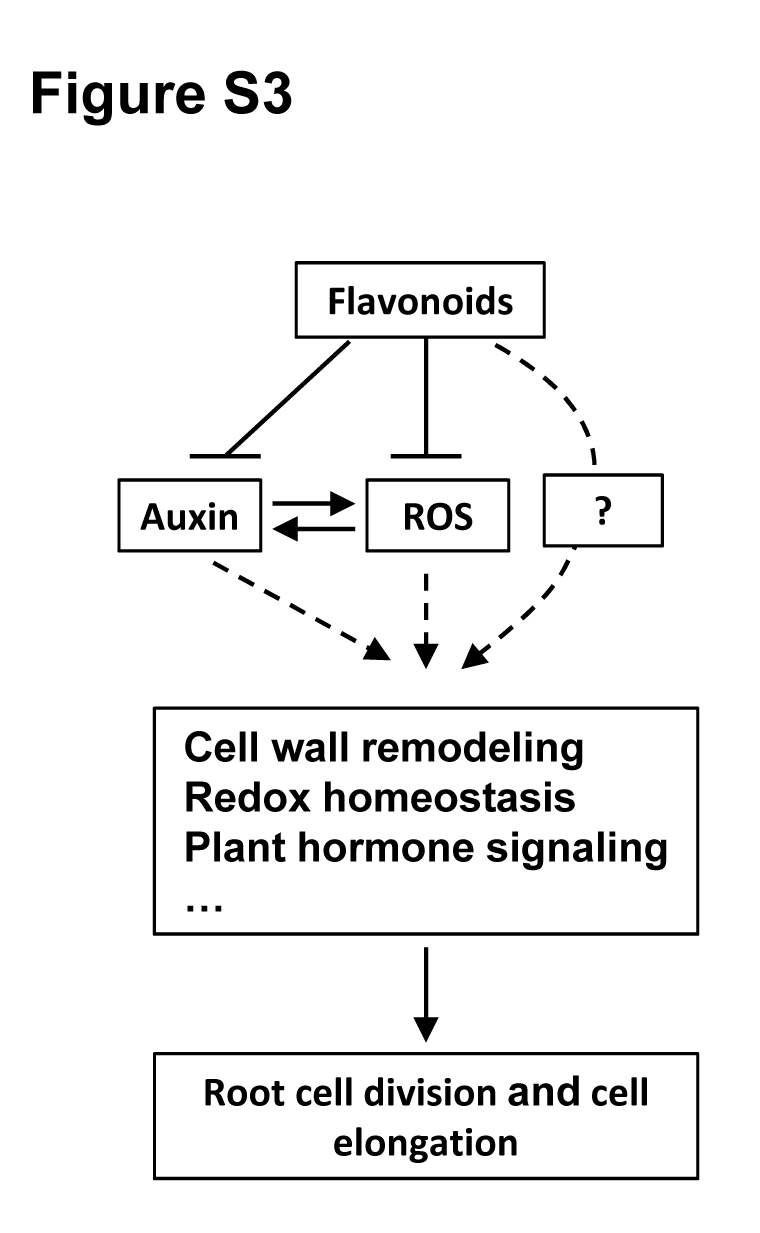

Supplement: Supplementary file 1 [file genes-10-00213-s001.zip › Xu et al-2019-Supplementary files v3/Figure S3.tif]
